# Immune Regulation of Tissue Repair and Regeneration via miRNAs—New Therapeutic Target

**DOI:** 10.3389/fbioe.2018.00098

**Published:** 2018-07-13

**Authors:** Celeste Piotto, Ziad Julier, Mikaël M. Martino

**Affiliations:** European Molecular Biology Laboratory Australia, Australian Regenerative Medicine Institute, Monash University, Melbourne, VIC, Australia

**Keywords:** microRNAs, regeneration, inflammation, immune system, biomaterials, neutrophils, macrophages, Tcells

## Abstract

The importance of immunity in tissue repair and regeneration is now evident. Thus, promoting tissue healing through immune modulation is a growing and promising field. Targeting microRNAs (miRNAs) is an appealing option since they regulate immunity through post-transcriptional gene fine-tuning in immune cells. Indeed, miRNAs are involved in inflammation as well as in its resolution by controlling immune cell phenotypes and functions. In this review, we first discuss the immunoregulatory role of miRNAs during the restoration of tissue homeostasis after injury, focusing mainly on neutrophils, macrophages and T lymphocytes. As tissue examples, we present the immunoregulatory function of miRNAs during the repair and regeneration of the heart, skeletal muscles, skin and liver. Secondly, we discuss recent technological advances for designing therapeutic strategies which target miRNAs. Specifically, we highlight the possible use of miRNAs and anti-miRNAs for promoting tissue regeneration via modulation of the immune system.

## Introduction

Tissue injury is followed by a cascade of processes leading to the restoration of tissue homeostasis and it is well recognized that the immune system is strongly involved in tissue healing. While numerous regulators are known to coordinate the immune response following injury, microRNAs (miRNAs) have emerged as important actors. Therefore, targeting the immunoregulatory roles of miRNAs could become an attractive option for regenerative therapies. miRNAs are well conserved among species and are involved in a variety of key biological processes by controlling post-transcriptional gene expression through decreasing mRNA stability and/or inhibiting translation. Currently, 1982 human miRNAs precursors are annotated into the miRBase (release 22), each of which are thought to regulate hundreds of target genes (Bartel, 2018; Zhang et al., 2018). Their small size and relatively long half-life (Marzi et al., 2016) make them attractive agents for clinical use. Moreover, a single miRNA can modulate numerous genes, thus having a stronger output than single gene therapy. Here, we first focus on the immunoregulatory role of miRNAs during tissue repair and regeneration. Then, we discuss various delivery systems and the potential targeting of miRNAs in immune cells to promote regeneration.

## Immunoregulatory role of miRNAs during tissue repair and regeneration

After injury, immune cells trigger a phase of acute inflammation that represents the first line of defense against pathogens. Moreover, inflammation and the immune response are critical to drive tissue repair and regeneration. Nevertheless, a sustained inflammation often impairs the healing process and its resolution is necessary to restore homeostasis. Various immune cell types are mobilized following tissue injury including neutrophils, monocytes/macrophages and Tcells (Julier et al., 2017; Larouche et al., 2018). Neutrophils are rapidly recruited to the injury site and promote monocyte recruitment which differentiate into macrophages. While pro-inflammatory macrophages (commonly named M1) maintain inflammation and initiate the first steps of tissue healing, anti-inflammatory macrophages (commonly named M2) contribute to resolve inflammation and promote tissue remodeling. Tcells are also important – for instance, pro-inflammatory macrophages are able to stimulate conventional Tcells in a positive-feedback loop, which in turn inhibit tissue repair via inflammatory cytokines or cytotoxic activity. In contrast, regulatory Tcells (Tregs) help maintaining an anti-inflammatory environment.

**Figure 1:**
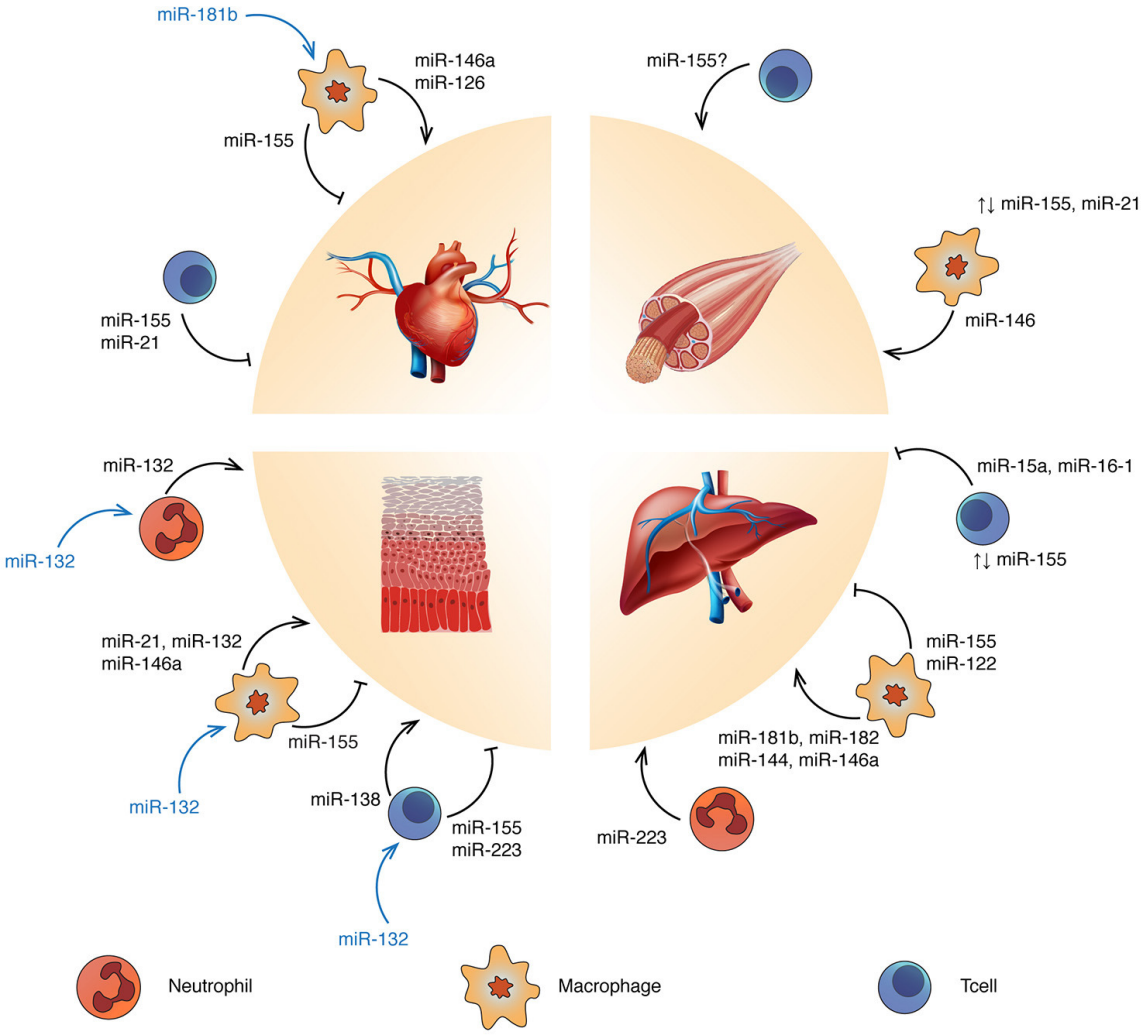
Targeting miRNA regulation to promote tissue repair and regeneration: ↑, overexpression; ⊥, inhibition; ↑↓, balanced expression. Blue and yellow cells represent Tcells and macrophages respectively. Refer to the text for the specific immune cell subtypes. miRNAs reported in blue refer to tissue-derived miRNAs affecting the related immune cell functions.

Because neutrophils, macrophages and Tcells are strongly involved in the tissue healing process, they are interesting targets for regenerative medicine. Activities of these immune cells can be modulated by delivering cells, cytokines and biomaterials. Alternatively, targeting miRNAs provides an interesting way to modulate the immune response following injury, since miRNAs are an endogenous mechanism to fine-tune gene expression. For instance, miR-21, miR-146a and miR-155 have been intensely investigated due to their involvement in Toll-like receptor (TLR) activation and inflammation. The anti-inflammatory miR-21 and miR-146a act in a negative feedback loop with the NF-kB pathway (Hou et al., 2009; Sheedy et al., 2010). By contrast, miR-155 is widely considered as a pro-inflammatory miRNA promoting interferon signaling (O'Connell et al., 2009). Other miRNAs are linked to the regulation of innate (Aalaei-andabili and Rezaei, 2013; He et al., 2014) and adaptive immunity (Baumjohann and Ansel, 2013; Tang et al., 2014; Liang et al., 2015). Moreover, immune cells communicate with each other or with neighboring cells by delivering miRNA-containing exosomes (Fernández-Messina et al., 2015). In the next sections, we discuss the immunoregulatory roles of miRNAs in the context of tissue repair and regeneration, highlighting examples in heart, skeletal muscle, skin, and liver (Figure 1).

### Heart

Mammalian adult heart cannot regenerate, due to the loss of cardiomyocytes proliferation capacity seven days after birth (Porrello et al., 2011). Indeed, myocardial infarction and other cardiovascular diseases leading to loss of tissue are frequently followed by fibrosis rather than the generation of new cardiomyocytes. Targeting the immune response after cardiac injury may improve current strategies, since excessive inflammation leads to further damage and infarct expansion. Macrophages are central regulators of cardiac repair (Fernández-Velasco et al., 2014; Fujiu et al., 2014) and affect fibroblast functions, which have a major role in fibrosis through extracellular matrix production (Takeda and Manabe, 2011). miR-155 has been associated with macrophage-induced cardiac hypertrophy (Heymans et al., 2013), inflammation and injury in viral heart disease (Corsten et al., 2012) and diabetic heart (Jia et al., 2017), by enhancing a pro-inflammatory phenotype. Furthermore, miR-155-enriched-exosomes from macrophages suppress fibroblast proliferation and enhance inflammation during cardiac injury (Wang et al., 2017a). Anti-inflammatory miRNAs such as miR-146a have been linked to improvement of cardiac function in sepsis mouse models (Gao et al., 2015). miR-146a reduces the expression of myocardial intercellular adhesion molecule 1 and vascular cell adhesion protein 1, thus decreasing infiltrating macrophages and neutrophils into the heart (Cavaillon and Adib-Conquy, 2005; Alves-Filho et al., 2008). Interestingly, cardiosphere-derived cells have demonstrated to have a role in macrophage polarization through exosome-mediated signaling. Exosomes secreted by cardiospheres delivered after myocardial infarction modulate macrophages toward a cardioprotective phenotype via miR-181b which is, at least partially, responsible for reduced proinflammatory signaling and enhanced phagocytosis (De Couto et al., 2017). The phagocytic activity of anti-inflammatory macrophages (efferocytosis) is likewise important for cardiac repair since it allows clearance of debris and apoptotic cardiomyocytes and neutrophils (Lörchner et al., 2015). In diabetic mouse models where efferocytosis is impaired, miR-126 overexpression rescues efferocytosis (Suresh Babu et al., 2016). This miRNA is also implicated in Treg regulation as it enhances Foxp3 expression, which drives Treg immuno-suppressive functions through interleukin (IL)-10 and transforming growth factor-β (TGF-β) production. While the role of Tcells in cardiac healing is becoming evident (Weirather et al., 2014), little is known about their regulation by miRNA in this context. Yet, in a mouse model of myocarditis, miR-155 drives Th17/Treg imbalance and its inhibition through a synthetic oligonucleotide (antagomir-155) demonstrated to be promising (Yan et al., 2016). Moreover, in patients carrying acute myocardial infarction, the number of circulating Tregs was lower and inversely correlated with miR-21, which seems to negatively regulate Tregs through the Foxp3-TGF-β1 axis (Li et al., 2015c).

### Skeletal muscle

Skeletal muscle regeneration is complex, relying on satellite cells (Chang and Rudnicki, 2014) along with immune cells (Bosurgi et al., 2011) and other cell types such as fibroblasts (Murphy et al., 2011). After injury, pro-inflammatory macrophages positively influence satellite cells and myoblast proliferation (Tidball, 2005; Arnold et al., 2007; Perdiguero et al., 2011). Then, anti-inflammatory macrophages induce myoblast differentiation/fusion and collagen production (Arnold et al., 2007). miR-155-deficient mice manifest delayed muscle regeneration, mainly due to unbalances between pro- and anti-inflammatory macrophages (Nie et al., 2016). This balance is also regulated by miR-21 which acts through the p38-miR-21-AKT pathway, which prevents anti-inflammatory polarization (Perdiguero et al., 2011). The importance of early inflammation controlled by miRNAs is confirmed in human where miR-146a expression, known to negatively regulate pro-inflammatory macrophage migration, is lower in polymyositis/dermatomyositis patients compared to normal subjects (Yin et al., 2016). Though many studies focused on macrophages, the role of Tcells in muscle regeneration has gained interest. For example, Burzyn and colleagues have demonstrated that Tregs enhance satellite cells colony-forming capacity and control Tcell infiltration, thus restricting the negative impact of T helper and cytotoxic Tcells on muscle repair. Treg accumulation in muscle coincides with the switch from pro- to anti-inflammatory macrophages (Burzyn et al., 2013) and IL-33 has been reported to be crucial for Tregs accumulation in muscle (Kuswanto et al., 2016). Interestingly, miR-155 appears to regulate IL-33 responsiveness in type 2 innate lymphoid cells (Johansson et al., 2017), but a direct link between miRNA regulation of Tcell functions and muscle regeneration has not yet been established.

### Skin

Scarring is a common feature of wound healing and the immune system is strongly involved in this process (Larouche et al., 2018). For example, mice lacking pro-inflammatory macrophages during the first/mid-stages of wound healing show impaired repair (Lucas et al., 2010). Nevertheless, polarization of macrophages to an anti-inflammatory phenotype is needed to continue the healing process. miR-146a, miR-132, and miR-21 have been shown to be important for wound healing immune regulation. For instance, miR-132 is up-regulated during the inflammatory-to-proliferative transition phases where it is mostly induced in keratinocytes, promoting their growth and decreasing chemokines production (Li et al., 2015a). Along with miR-146a, miR-132 promotes an anti-inflammatory macrophage polarization (Essandoh et al., 2016) and is highly expressed in skin-infiltrated neutrophils (Larsen et al., 2013). miR-146a contributes to inflammation resolution acting mainly through keratinocytes. This miRNA is up-regulated upon injury via the NF-kB pathway and its long-lasting expression is necessary to down-regulate keratinocytes inflammatory cytokines expression (IL-17, IL-8, and TNF-α) (Meisgen et al., 2014; Srivastava et al., 2017). miR-21 regulates efferocytosis in macrophages, where its expression level increases after apoptotic neutrophil engulfment (Das et al., 2014). miR-21 is found overexpressed in infiltrating T helper cells in skin psoriasis, but in this context, it may contribute to inflammation by supporting Tcell survival (Meisgen et al., 2012). Further evidences regarding Tcell regulation via miRNA during skin healing principally come from two studies. miR-155 and miR-223 have been shown to worsen psoriasis conditions, potentiating Tcell response and Th17-IL-17 production (Løvendorf et al., 2015), while high level of miR-138 could ameliorate skin healing through Th2-IL4 (Fu et al., 2015).

### Liver

The liver possesses a high regenerative ability and can regenerate after a partial hepatectomy (Michalopoulos, 2017). However, this capacity does not improve the outcome of many liver diseases, particularly those associated with chronic inflammation, leading to fibrosis and cirrhosis (Marcellin and Kutala, 2018). miR-122 is a liver-specific miRNA accounting for more than half of all liver miRNA species. The miRNA is abundant in hepatocytes where involved in hepatic functions and diseases (Otsuka et al., 2017) and intercellular communication through exosomes. High levels of miR-122-enriched exosomes are present in the sera of mice and healthy individuals after alcohol consumption or chronic consumption. miR-122-enriched exosomes are horizontally transferred to monocytes, sensitizing them to lipopolysaccharide and inducing inflammatory reactions (Momen-Heravi et al., 2015). Other miRNAs involved in alcoholic liver disease (ALD) progression through immune cells modulation are miR-181b, miR-155 and miR-223. miR-181b and miR-155 are, respectively, down-regulated and up-regulated in Kupffer cells of ALD mouse models resulting in sensitization of inflammatory pathways through NF-kB signaling (miR181b) (Saikia et al., 2017) and TNF-α mRNA stabilization (miR-155) (Bala et al., 2011). miR-223 acts in neutrophils, by inhibiting the oxidative stress pathway and thus reducing injury exacerbation and fibrosis by reactive oxygen species (Li et al., 2017). Decreasing the pro-inflammatory miR-155 has proven to positively affect non-alcoholic diseases. miR-155 deficiency in ischemia-reperfusion injury mice induces the development of anti-inflammatory macrophages (Tang et al., 2015), while miR-182 and miR-146a overexpression protects the liver by inactivating TLR4 pathway (Jiang et al., 2014, 2016). In non-alcoholic steatohepatitis, TLRs play important roles (Roh and Seki, 2013) and it has been demonstrated that TLR2-enhanced expression of Nod-like receptor protein 3 via NF-kB, promotes NLRP3-inflammasome activation in concert with saturated fatty acid in Kupffer cells (Miura et al., 2013). A study in a mouse model of non-alcoholic steatohepatitis identified decreased miR-144 expression in Kupffer cells with consequent induction of TLR2 (Li et al., 2015b). Regarding the immunoregulatory role of miRNA in Tcells, two studies that used a concanavalin A-treated mice model of liver injury revealed miR-155 and miR15a/16-1 as potential miRNA targets to restore liver homeostasis. Blaya et al. found an altered miR-155 expression in both liver and peripheral blood mononuclear cells, with significant lower Treg recruitment in *miR-155*^−/−^ mouse (Blaya et al., 2018), while Lu et al. demonstrated that the deletion of miR15a/16-1 in CD4^+^ cells promotes liver regeneration through IL-22 up-regulation (Lu et al., 2018).

## Potential miRNA and anti-miRNA delivery systems to promote tissue regeneration via immunoregulation

miRNA are emerging as a novel therapeutic approach and numerous miRNAs have reached clinical trials for the treatment of various diseases from cancer (van Zandwijk et al., 2017) to regenerative medicine (Curtin et al., 2018) (Table 1A). Therapeutic miRNA agents include a wide range of miRNA modifications, mostly regarding miRNA inhibition strategies (Table 1B). In addition, many delivery methods have been developed and tested in animal models (Table 1C).

**Table 1A:** miRNA in pre-clinical and clinical trials for regenerative medicine.

**Agent**	**Method of administration**	**Therapeutic effect**	**Clinical trial**	**BioPharmaceutical company**	**References**
					
*miR-29 mimics* MRG-201	Direct skin injection	Anti-fibrous scar formation	Phase I	miRagen Therapeutics	ClinicalTrials.gov identifier: NCT02603224
*LNA-anti-miR-208* MGN-9103	Intravenous injection	Treatment of chronic heart failure, preventing hypertrophy, fibrosis and pathological remodeling	Pre-clinical trial	miRagen Therapeutics	Montgomery et al., 2011; Eding et al., 2017
*LNA-anti-miR-15 family* MGN-1374	Intravenous injection	Post-myocardial infarction remodeling, enhances cardiomyocytes proliferation	Pre-clinical trial	miRagen Therapeutics	Hullinger et al., 2012
*2′Ome anti-miR-21* RG-012	Subcutaneous injection	Alport syndrome, decreases renal fibrosis progression	Phase II	Regulus Therapeutics	ClinicalTrials.gov identifier: NCT02855268
*2′MOE, 2′fluoro-sugar modified nucleosides anti-miR-155*	Intraperitoneal injection	Amyotrophic lateral sclerosis	Preclinical trial	Regulus Therapeutics	Koval et al., 2013

**Table 1B:** miRNA modifications to achieve miRNA inhibition or upregulation, both *in vitro* and *in vivo*.

**Strategy**	**Synthesis**	**Characteristic**	**Advantages**	**Disadvantages**	**References**
					
**miRNA inhibition**	AMO	Ribose 2′ hydroxyl group methylation (OMe)	Higher RNA binding affinity, little improvement in nuclease resistance	Poor stability in serum	Esau, 2008; Lennox et al., 2013
	Antagomirs	2′-OMe, 3′-end conjugated cholesterol	Nuclease resistance, crossing of plasma membrane without delivery vectors	High doses required, *in vivo* off-targets	Krützfeldt et al., 2005, 2007; Rebustini et al., 2016
	LNA	Ribose 2′-O:4′-C methylene bridge	Highly resistant to nuclease, lower doses required (compared to antagomir)	Possible off-targets	Mook et al., 2010; Obad et al., 2011
	PMO	Substitution of ribose (6-morpholine rings) and phosphodiester bonds (phosphorodiamidates)	Neither nuclease nor enzymatic degradation	Lower binding affinity to miRNA	Warren et al., 2012
	PNA	Synthetic DNA analog, repeated units of N-(2-aminoethyl) glycine linked by peptide bonds	Neither nuclease nor enzymatic degradation, high DNA/RNA binding affinity and specificity	Poor uptake by cells	Nielsen, 1999; Oh et al., 2009
	miRNA sponge	Plasmid encoding transcript with multiple competitive miRNA binding sites	Longer expression, ideal for chronic disease	High miRNA concentration needs strong promoters or multiple vector copies for miRNA inhibition, high sponge expression level leads to off-targets	Ebert and Sharp, 2010; Tay et al., 2015
**miRNA replacement**	Mimics	Artificial double-stranded RNA	Directly loaded into the RISC	Higher degradation in biological fluids, possible dose-related off-targets	Wang, 2009

*AMO, Anti-miRNA oligonucleotide; LNA, locked nucleic acid; PMO, Phosphorodiamidate morpholino oligonucleotide; PNA, peptide nucleic acid*.

**Table 1C:** miRNA and anti-miRNA delivery strategies.

**Method of delivery**	**Advantages**	**Disadvantages**	**References**
			
**Direct injection**	Easiest method, lower doses required	Limited access to certain tissues/organs, rapid clearing by kidneys	Frith et al., 2014
**Viral based methods**	Long-term/inducible expression of transgene, high transfection efficiency	Inherent toxicity and immunogenicity, possible mutagenic insertion	Frith et al., 2014; Gori et al., 2015
**Non-viral or synthetic methods**	Lower toxicity and immunogenicity, lower cost and higher versatility (compared to viral methods)	Less efficiency (compared to viral methods)	
			
Cationic Liposomes	Protect RNA from nucleases increase circulation half-life, lower degree of genetic perturbation	Cytotoxicity; poor *in vivo* stability and reproducibility	Gori et al., 2015; Peng et al., 2015
Exosomes	Biocompatibility, stability in the circulation, biological barrier permeability, specific targeting upon engineering with recognition factor, low immunogenicity, low toxicity	Contents not fully characterized, could aggravate present disease or tumor depending on their source of isolation	Bjørge et al., 2018
Cationic Polymer Vectors *(synthetic and natural)*	High flexibility (weight, molecular structure, composition, stimuli-sensitivity), low toxicity and immunogenicity, high transfection efficiency	Synthetic: often poorly biodegradable and toxic (PEI), accumulation in the liver (PAMAMs) Natural: biodegradability in sera (CPPs)	Gori et al., 2015; Peng et al., 2015; Yang, 2015
Nanoparticles	Non-immunogenic, most are non-toxic, less susceptible to nucleases, greater cellular uptake	Toxicity of some metal NP, possible agglomeration, possible cause of inflammation	Fu et al., 2014; Gori et al., 2015; Fernandez-Piñeiro et al., 2017
**Scaffold-based methods**	Controlled, localized and prolonged transgene expression, combination with stem cells and other therapies, offers protection from immune response to viral or non-viral miRNA delivery methods when combined	Possible immune reaction with natural scaffold, possible miRNA inactivation during sterilization process (avoided with miRNA immobilization directly onto the scaffold surface after sterilization)	Gori et al., 2015; Peng et al., 2015
**Cells as delivery vehicles** *(MSCs, mostly used)*	Naturally migrate to the injured area, have immuno-suppressive properties, influence both ECM and other cells through factors release and miRNA-EVs, can be genetically engineered with selected miRNA mimics	The large number of required MSCs needs *in vitro* expansion that may result in mutations accumulation, MSCs could support undiagnosed tumor, difficulties in brain homing, difficulties in tracking all single MSCs to control proper homing to target tissue, origin tissue microenvironment affects stem cell functions	Gori et al., 2015; Sherman et al., 2015

*MSC, mesenchimal stem cells; EV, extracellular vesicles; ECM, extracellular matrix; NP, nanoparticles; PEI, Polyethylenimines; PAMAMs, poly-amidoamines; CPP, cell penetrating peptide*.

While direct injections of miRNAs or miRNA inhibitors have been widely used, this simple approach present limitations such as *in vivo* stability and biodistribution. The development of advanced delivery systems could overcome direct injection limitations (Zhang et al., 2013; Frith et al., 2014). Among delivery systems, exosomes have been explored to some extent, because they are the natural delivery system of miRNA *in vivo*. They protect miRNAs from degradation during systemic transport and can target specific cell types through membrane ligands. In addition, although they carry MHC-I and/or MHC-II peptides, allogenic exosomes have low immunogenicity. Their cargo composition is tightly regulated, but can change according to specific microenvironment conditions and this may lead to unwanted miRNA species. The main obstacles are the low amount of exosomes secreted by mammalian cells and the complex process of purification, which limit their usage in regenerative medicine. Artificial mimetic exosomes have the potential to overcome these disadvantages and avoid possible immune responses. The challenge is to engineer mimetic exosomes with components of natural exosomes that are still not well defined (Barile and Vassalli, 2017; Bjørge et al., 2018; Kim et al., 2018). Among artificial lipid-based vectors, liposomes are extensively used for *in vitro* application. Cationic vesicles share the advantages of exosomes and bind miRNA/anti-miRNA molecules via electrostatic interactions. Their composition can be modified to mimic exosomes, but they usually exhibit higher toxicity than their natural counterpart and can activate the complement (Szebeni, 2005; Gori et al., 2015; Peng et al., 2015). For instance, locked nucleic acid (LNA)-based anti-miR-21 and anti-miR-712 have been delivered in mouse models of atherosclerosis and nerve trauma, through liposomes or cationic lipids-coated nanoparticles (NPs), to reduce the inflammatory macrophage number (Kheirolomoom et al., 2015; Simeoli et al., 2017).

In contrast to liposomes, NPs may show less immunogenicity issues. NPs present various advantages such as small size (10–1,000 nm), high surface area, good stability in physiological media and great cellular uptake. Inorganic NPs are made of various solid materials such as gold, silicon, magnesium, silver, and iron (Gori et al., 2015; Ahmadzada et al., 2018). For example, two recent studies have used gold NPs as carriers of miRNAs mimics and antagomirs to promote mouse osteogenic differentiation and osseointegration of implants (Liu et al., 2017b; Yu et al., 2017). Organic NPs are lipid-, proteic-, or polymer- based and have been exploited in clinical studies. Cationic polymer-based NPs made of natural/synthetic polymers have a great binding affinity for miRNA and are frequently complexed with hyaluronic acid (HA), which enhances biocompatibility and gene transfection efficiency. HA-chitosan-based NPs have been used in two studies to promote osteogenesis through *in vitro* miRNA mimics and *in vivo* antagomirs transfection, in human and mice mesenchymal stem cells respectively (Wang et al., 2016; Wu et al., 2016). As another example, hyaluronic acid-poly(ethylenimine)-based NPs or polymer complexes have been used in mice to deliver miR-125 and miR-155 into macrophages (Liu et al., 2017a; Parayath et al., 2018).

As an alternative to these delivery systems, scaffold-mediated delivery is particularly interesting in the context of tissue regeneration. The scaffold can be delivered directly in the injured site and can naturally contain or be functionalized with additional pro-regenerative molecules (Peng et al., 2015; Curtin et al., 2018). For instance, a single injection of HA hydrogel-miR-302 mimics complexes in mice hearts after ischemic injury promotes cardiomyocytes proliferation (Wang et al., 2017b). As other examples, a porous collagen-nanohydroxyapatite scaffold containing the antogomir-133a has been used to promote bone regeneration via mesenchymal stem cell-mediated osteogenesis (Menciá Castanõ et al., 2016), and a polymer-based scaffold (polyethylene glycol and polyethylenimine) has been used for miR-26 mimics delivery to promote angiogenesis and osteoclast formation (Zhang et al., 2016).

## Future directions

Targeting miRNA still presents limitations. The complex regulatory network of a single miRNA renders the precise identification of all its target difficult, leading to possible unwanted mRNA silencing. Dosage is also critical—low level of miRNA may be insufficient to achieve the desired outcome, while high level of miRNA is likely to hold the RNA-induced silencing complex (RISC), preventing the action of endogenous miRNAs and leading to off target consequences. This is a problem in common with anti-miRNA therapy, since mature miRNAs are bound to RISC proteins and their inhibition does not allow the release of the complex. Most anti-miRNA molecules are perfectly complementary to the *seed region* of miRNAs and are chemically modified to increase the melting temperature of the anti-miRNA-miRNA complex. However, the inhibitory interaction under physiological conditions is less strict and anti-miRNAs are frequently unable to distinguish among miRNAs of the same family. An alternative anti-miRNA method could act at precursor stages of miRNA maturation. The longer sequence of primary (>1,000 nt) and precursor (~70 nt) miRNAs contains non-conserved regions that differ even among miRNAs of the same family. Although this strategy may be a viable approach to overcome cross-family miRNA inhibition, further efforts are necessary to develop diverse anti-miRNA species since most of them concentrate on mature miRNAs.

Examples of systems that specifically deliver miRNAs/anti-miRNAs to immune cells are still sparse. The use of ligands, peptides or antibodies with nanotechnology allow targeting specific cells. For instance, lipid nanoparticles coated with a single-chain antibody specific for a dendritic cell receptor enable the release of siRNAs to a subset of dendritic cells (Katakowski et al., 2016). Interesting nanocarriers are for example nucleic acid aptamers, single-stranded DNA or RNA oligonucleotides that bind the target molecule with high affinity. They are remarkably stable, present a very low immunogenicity and allow extensive site-specific chemical modification, offering a wide range of targets such as proteins, nucleic acids, carbohydrates or whole cells (Zhou and Rossi, 2018). For example, an aptamer-siRNA conjugate has been developed to target Tcells, thus releasing anti-HIV siRNAs in HIV-infected mice (Zhou et al., 2013). This approach to engineering miRNA-carriers that target specific immune cells could be used for regenerative medicine applications.

In conclusion, further advances are necessary to better understand the complex regulatory network of miRNAs and to predict the outcome of miRNA replacement or inhibition *in vivo*. The development of novel and specific miRNA delivery strategies to immune cells could create new opportunities to promote tissue regeneration via immune regulation.

## Author contributions

CP, ZJ, and MM wrote the manuscript. CP made the figure and table. MM supervised the writing.

### Conflict of interest statement

The authors declare that the research was conducted in the absence of any commercial or financial relationships that could be construed as a potential conflict of interest.
